# The role of Nrf2 signaling pathway in diabetic cardiomyopathy: from pathogenesis to traditional Chinese medicine interventions

**DOI:** 10.3389/fcvm.2025.1492499

**Published:** 2025-07-14

**Authors:** Huixian Gu, Junchang Liu, Jie Jiang, Hongyan Ma, Siyu Wu, Longfei Sun, Lan Yao

**Affiliations:** ^1^ College of Traditional Chinese Medicine, Xinjiang Medical University, Urumqi, China; ^2^ Xinjiang Uygur Autonomous Region Hospital of Traditional Chinese Medicine, Urumqi, China; ^3^ Xinjiang Key Laboratory of Famous Prescription and Science of Formulas, Xinjiang Medical University, Urumqi, China

**Keywords:** Nrf2 signaling pathway, diabetic cardiomyopathy, pathogenesis, traditional Chinese medicine, therapeutic mechanism

## Abstract

Diabetic cardiomyopathy (DCM), a common diabetic complication independent of hypertension, coronary heart disease and heart valve disease is a major cause of cardiovascular mortality. The pathogenesis of DCM is complex. The Nrf2-related signaling pathway which regulates oxidative stress, energy metabolism and mitochondrial physiology may play important role in the pathogenesis of DCM. Current treatments for DCM focus on blood glucose and pressure control, cardiovascular protection, lipid lowering and blockade of the renin-angiotensin system. However, the adverse drug reactions are inevitable. Traditional Chinese medicine (TCM) as a multi-target, multi-pathway treatment approach is considered to be a promising therapy for DCM. We reviewed how the Nrf2 and related signaling pathway regulated pathophysiological processes such as oxidative stress, inflammation, myocardial fibrosis, apoptosis, ferroptosis, autophagy and mitochondrial dysfunction in the progression of DCM and explored the potential mechanism and clinical value of TCM in DCM treatment. Based on a literature review, we found that various herbal compounds and combinations could alleviate DCM via the Nrf2 signaling pathway. This review highlighted the role of the Nrf2 signaling pathway in DCM progression and put forward new therapeutic strategies for DCM.

## 1 Introduction

Diabetic cardiomyopathy (DCM) is a common complication of diabetes mellitus (DM) (1). It is a pathophysiological state characterized by metabolic disorders and microvascular lesions, which can lead to subclinical cardiac dysfunction, including left ventricular fibrosis, diastolic dysfunction and ultimately the heart failure (HF). In other words, cardiovascular disease is a major cause of death in diabetic patients. However, DCM as a unique disease is independent disease from traditional HF risks such as hypertension, coronary heart disease and valvular heart disease (2).

During the initial stage of DCM, metabolic disorders manifest as the impaired insulin metabolic signaling, the increased uptake of myocardial free fatty acids (FFAs) and mitochondrial dysfunction. These factors can worsen myocardial fibrosis, accelerate cardiac remodeling and ultimately reduce the ejection fraction (EF) in diabetic patients (3). In the later stages of DCM, there are more noticeable changes in cardiac structure, including myocardial cell necrosis, collagen accumulation, increased cross-linking of connective tissue, myocardial interstitial fibrosis and myocardial hypertrophy (4). With the increasing number of diabetic patients in China, the incidence of DCM has also risen rapidly (5). Currently, there is no specific therapy for DCM. Drug interventions mainly focus on blood glucose and pressure control, cardiovascular protection, lipid lowering and blockade of the renin-angiotensin system (2, 6). Unfortunately, the development and progression of cardiomyopathy in the patients with diabetes remains unrecoverable due to a poor prognosis (6). Additionally, the pathogenic factors of DCM can be proposed as the hyperlipidemia, inflammatory cytokines, oxidative stress, mitochondrial dysfunction and programmed cell death (7). However, the precise pathogenic mechanisms of these pathogenic factors in DCM still need to be clarified which is attractive for DCM therapy.

The Nrf2 signaling as one of the most critical intracellular signaling pathways and antioxidant defense systems controls essential cellular function including, but not limited to, cell proliferation, metabolism and extracellular matrix (ECM) remodeling (8, 9). Nrf2 belongs to the Cap “n”collar (CNC) transcription factor family and consists of multiple homologous domains, each one with a different functions (10). Under normal physiological conditions, Nrf2 binds to its inhibitor Keap1 in the cytoplasm, which facilitates the rapid ubiquitination and subsequently degradation of Nrf2 by the proteasome (11). However, when cells experience oxidative stress, electrophilic compounds, Nrf2 is unaffected by Keap1 and directly translocates to the nucleus. In the nucleus, it binds to antioxidant response elements (AREs) found in genes encoding antioxidant enzymes such as nicotinamide adenine dinucleotide phosphate (NADPH), quinone oxidoreductase (NQO1), glutathione S-transferase (GST), heme oxygenase-1 (HO-1), and γ-glutamyl cysteine synthase (*γ*-GCS) to increase the expression of AREs which play the vital role of detoxification, antioxidant and anti-inflammatory effects (12, 13). Studies have confirmed that chronic hyperglycemia not only generates extra reactive oxygen species (ROS) but also impairs antioxidant capacity orchestrated by downregulation of Nrf2 in the heart (14). The molecule agonists targeting key kinase components of the Nrf2 signaling pathway have drawn extensive attention and have been developed and evaluated in preclinical models of DCM (15). Therefore, further understanding of characterization and regulatory mechanisms governing abnormal regulation of Nrf2 signaling in DCM from current literature researches will provide important insights into possible future directions for targeted therapeutic regimen and a new combinatory therapeutic approach for DCM.

Currently, the conventional treatment of DCM drugs include metformin, thiazolidinediones (TZDs), sulfonylureas, Glucagon-like peptide-1 receptor (GLP-1R) agonists, dipeptidyl peptidase-4 (DPP-4) inhibitors, SLGT-2 inhibitors and angiotensin-converting enzyme inhibitors (ACEI). Unfortunately, some contraindications and side effects from these drugs are inevitable on account of a long-time treatment (2, 16). Traditional Chinese medicine (TCM) has its own unique diagnosis and treatment system, which has been used in clinical treatment for more than 2000 years in Chinese history (17). Furthermore, TCM has been widely utilized in the treatment of DCM among clinicians in China (18). Increasing studies confirm that TCM ameliorate DCM through the synergistic benefits of its multiple components and multiple targets, which involves various signaling pathways (19). Compared to the conventional therapies, TCM is characterized by multiple targets, multiple pathways, fewer side effects and greater accessibility. It is considered to be a valuable and effective therapeutic way for chronic metabolic diseases such as DCM (20, 21). Beyond their direct protective effects on the heart, TCM can also assist in lowering blood glucose and lipid levels, thus indirectly reducing the metabolic burden on myocardium (22, 23). Besides, TCM emphasizes evidence-based and individual-based treatment, which can adjust the drug regimen according to the patient's physical situation in order to reduce unnecessary side effects (24, 25). What is noteworthy is that Nrf2 signaling is regarded as a key therapy targets based on TCM in preventing the progression of DCM (14).

In this review, we firstly summarized the characteristics of Nrf2 signaling participation in different pathogenic factors for DCM. Furthermore, we investigated up-to-date activators of the Nrf2 signaling pathway for DCM treatment, including compounds from TCM and TCM formulations as well as their characteristics on therapeutic mechanisms in order to provide valuable and effective direction for DCM therapy.

## 2 Correlation between the Nrf2 signaling pathway and diabetes cardiomyopathy

The pathogenesis of DCM is considered to involve complex interactions among multiple factors. Nrf2 as a key transcription factor plays a role in the progression of DCM by regulation of mitochondrial dysfunction, reactive oxygen species (ROS) production, apoptosis, inflammatory cytokines secretion, myocardial fibrosis, ferroptosis and autophagy.

### 2.1 The role of the Nrf2 signaling pathway in oxidative stress induced by DCM

**Figure 1:**
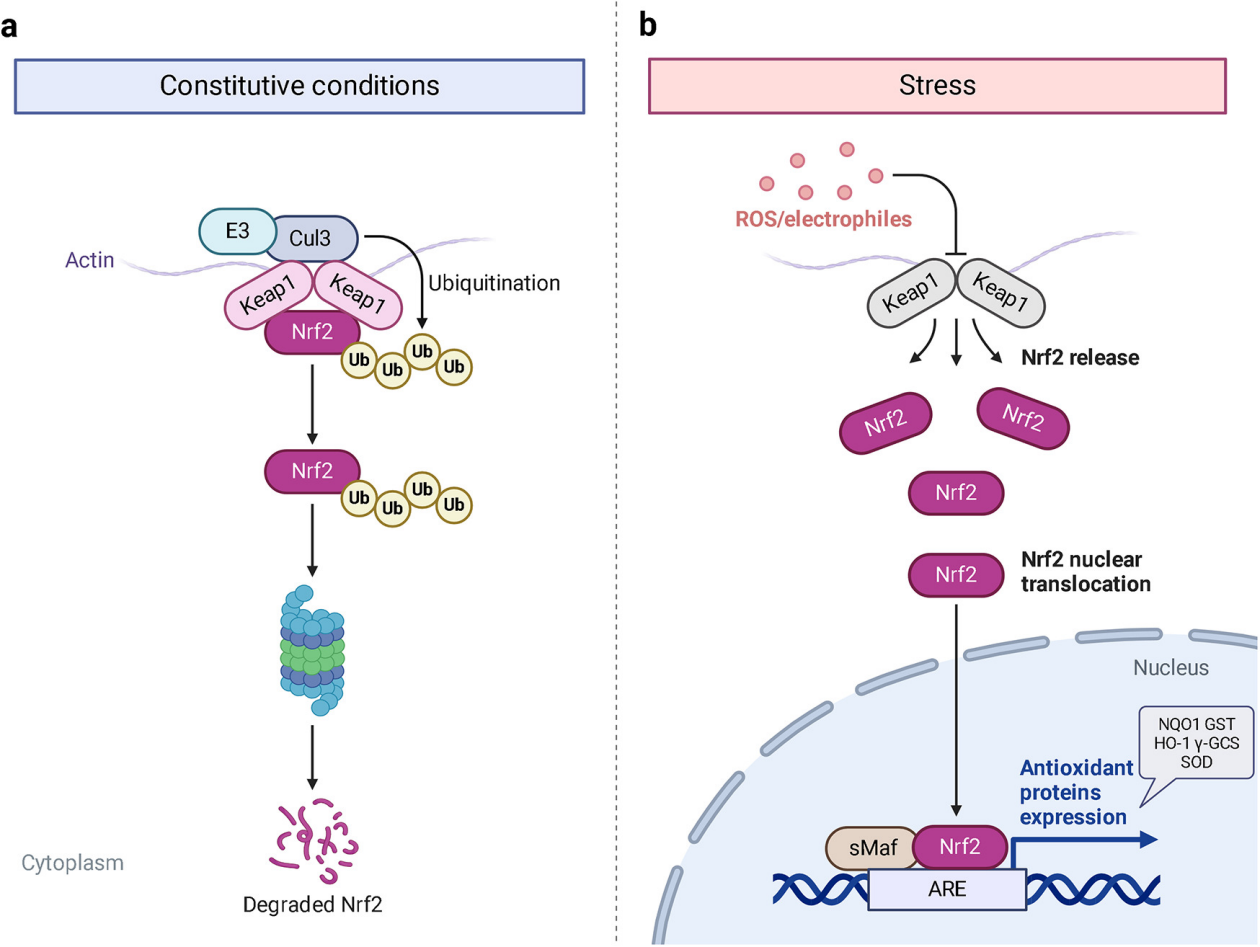
The process of Keap1 regulating Nrf2. E3 and Cul3 are ubiquitin ligases. **(a)** Normal physiological condition. **(b)** Oxidative stress condition.

Hyperglycemia stimulates the excessive production of ROS which impairs the endogenous antioxidant system and leads to oxidative stress in cardiomyocytes. As a results, inhibition of oxidative stress can improve heart function in patients with diabetes (26). Under normal physiological conditions, Nrf2 binds to Keap1 to form a stable complex. However, under oxidative stress, Nrf2 is released from Keap1 with phosphorylation and translocation into the nucleus. The phosphorylated Nrf2 then binds to the ARE and activates the transcription of antioxidant genes (27, 28) (Figure 1). PI3K/Akt pathway is also involved in Nrf2 activation and nuclear translocation. In doxorubicin (Dox)-induced H9c2 cardiomyocytes, activation of the PI3K/Akt signaling pathway upregulates the Nrf2 expression, which subsequently increases the protein expression of HO-1, NQO-1, and SOD and reduces oxidative stress (29). Studies have shown a significant downregulation of Nrf2 in both animal models of diabetes and diabetic patients' hearts which may be cause of angiogenic abnormalities, endothelial dysfunction and myocardial damage (14). While the upregulation of Nrf2 expression can protect cardiomyocytes from hyperglycemic injury (30). Priclincal experiments has confirmed that in the cardiomyocytes of streptozotocin (STZ)-induced diabetic rats and high glucose-induced H9c2 cells, the expression of Nrf2 is significantly downregulated. On the contrary, activation of Nrf2 can stimulate its downstream target genes expression such as SOD, HO-1, and NQO-1, thereby improving cardiomyocyte damage caused by oxidative stress, apoptosis and left ventricular dysfunction in DCM rats (31).

### 2.2 Nrf2 signaling pathway participates in the inflammatory response caused by DCM

During the progress of inflammatory responses in DCM, cellular events are closely linked to redox balance (32). Metabolic disorders resulting from diabetes trigger the production of inflammatory factors such as IL-6, TNF-α and monocyte chemoattractant protein-1 (MCP-1). These factors, in turn, activate the NF-κB and Toll-like receptor-mediated inflammatory pathway and stimulate inflammatory cell infiltration (e.g., macrophages and neutrophils) which leads to myocardial inflammation. This process significantly damages cardiomyocytes and exacerbates the progression of DCM (33–35). Between the Nrf2 and NF-κB signaling pathways, there exists a complex and dynamic interplay. Both of the pathways modulate cellular redox homeostasis and mediate responses to stress and inflammation (36). The Nrf2 as an upstream inhibitor reduces intracellular ROS production, thereby inhibiting proinflammatory signaling. Researchers have shown that activation of Nrf2 signaling controls the redox balance that it exerts on inflammatory networks (37). Activation of the Nrf2/HO-1/NF-κB signaling pathway can reduce cardiomyocyte inflammation injury caused by ischemia-reperfusion (38).

### 2.3 Activation of the Nrf2 signaling pathway inhibits myocardial fibrosis caused by DCM

Myocardial fibrosis and collagen deposition represent the critical structural modifications observed in DCM (39). The transition from cardiac fibroblasts (CFs) to myofibroblasts is a crucial cellular event in myocardial fibrosis (40, 41). TGF-β1 is a well-known inducer involved in the differentiation of CFs into myofibroblasts (42). It has been demonstrated that hyperglycemia can upregulate the expression of TGF-β1 in CFs (43), thereby promoting myocardial fibrosis and impairing the compliance of cardiac tissue in diabetic patients (26). Studies have confirmed that the activation of Nrf2 signaling pathway plays a beneficial role in the development of myocardial fibrosis under hyperglycemic conditions involves inhibition of various redox signaling elements such as TGF-β1, profibrogenic genes, cardiac remodeling-associated lncRNAs (44, 45). Preclinical experiment indicates that activation of the Nrf2/HO-1 signaling axis inhibits the TGF-β1/Smad2/3 signaling pathway, effectively suppressing myocardial fibrosis in the DCM mice model (46, 47). The primary crosstalk mechanisms between Nrf2 and TGF-β1 are as follows. Firstly, Nrf2 reduces MMP-9 expression, which in turn decreases the levels of TGF-β1. Secondly, Nrf2-mediated Smads inhibition is associated with increased Smad7 levels, negatively modulating factor of the TGF-β1 signaling pathway (48).

### 2.4 Nrf2 signaling pathway inhibits apoptosis caused by DCM

It has been confirmed that metabolic disorders in DCM can induce maladaptive cardiomyocyte apoptosis. This situation can be caused by hyperglycemia, mitochondrial damage and dysfunction, energy metabolic disturbance, excessive ROS, endoplasmic reticulum stress (ERS), advanced glycation end products (AGEs) and inflammation (49, 50). Long-term hyperglycemia in diabetic patients can lead to cardiac contractile dysfunction and remodeling, attributed to cardiomyocyte apoptosis (51). Under hyperglycemic conditions, oxidative stress caused by ROS is heightened and may contribute to cardiomyocyte apoptosis (52, 53). Studies have shown that Nrf2 as an antioxidant response element plays a vital role in preventing ROS-induced cell apoptosis in both vasculature and heart tissue (54). In STZ-induced diabetic rat model, cardiomyocyte apoptosis was observed along with decreased protein expression of Nrf2 as well as its downstream antioxidant enzymes HO-1 and *γ*-GCS. Blocking the protein expression of Akt, Nrf2, HO-1, *γ*-GCS, and caspase3 by PI3K-specific siRNA and a PI3K inhibitor of LY294002 exacerbates high glucose-induced oxidative stress and cardiomyocyte apoptosis. These results suggest that the PI3K/Akt/Nrf2/HO-1 signaling pathway may play a significant role in the antioxidative effect of DCM (55). Additionally, overexpression of miR-155 in H9c2 cells induced by high glucose leads to decreasing expression of endonuclear Nrf2 and HO-1 which accompanied by the cell apoptosis (56). Furthermore, study demonstrates that activating the Nrf2-related signaling pathway alleviates cardiomyocyte apoptosis in DCM mice model (57).

### 2.5 Nrf2 signaling pathway inhibits ferroptosis caused by DCM

Ferroptosis is a form of cell death characterized by the accumulation of lipids and lipid peroxidation (58). Under conditions of metabolic disorder, cardiomyocytes predominantly derive energy from fatty acid oxidation, which induces excessive fatty acid oxidation and the accumulation of peroxides and inflammatory factors, ultimately resulting in ferroptosis and irreversible damage to cardiomyocytes (59). However, the activation of Nrf2 stimulates the expression of numerous antioxidant factors, such as HO-1 and glutathione peroxidase 4 (GPX4), thereby inhibiting the progression of ferroptosis (60). Research has shown that activation of the Nrf2/GPX4/glutathione (GSH) pathway leads to increased SOD levels and downregulation of both MDA and free ferrous iron (Fe^2+^) which effectively mitigates oxidative stress and ferroptosis in high glucose-induced H9c2 cells (61, 62). Besides, the cystine/glutamate antiporter SLC7A11 as one of the key regulators of ferroptosis is a downstream target of Nrf2. In cardiomyocytes of DCM mice model, activation of the AMPK/Nrf2 pathway can reverse the decrease expression of SLC7A11 and GSH levels and consequently balance iron metabolism (63).

### 2.6 The role of the Nrf2 signaling pathway in autophagy caused by DCM

Autophagy as an adaptive response can help cells to cope with various stresses, including hyperglycemia, hypoxia, oxidative stress, and exogenous stress (64). Autophagy occurs in almost all types of cardiovascular cells (65). Prolonged hyperglycemia can disrupt cardiomyocyte autophagy (66), exacerbating the progression of DCM. Nrf2, a gene with potential antioxidant functions, is capable of regulating autophagy through positive effect. Studies have shown that activation of the Nrf2 signaling pathway increases autophagy and alleviates high glucose-induced hypertrophy of H9c2 cells (67). Activation of the PP2A/Nrf2 signaling pathway can promote cardiomyocyte autophagy under high glucose condition (68). However, prolonged activation of Nrf2 may suppress autophagy via a non-canonical mechanism (69).

### 2.7 The role of the Nrf2 signaling pathway in mitochondrial dysfunction caused by DCM

**Figure 2:**
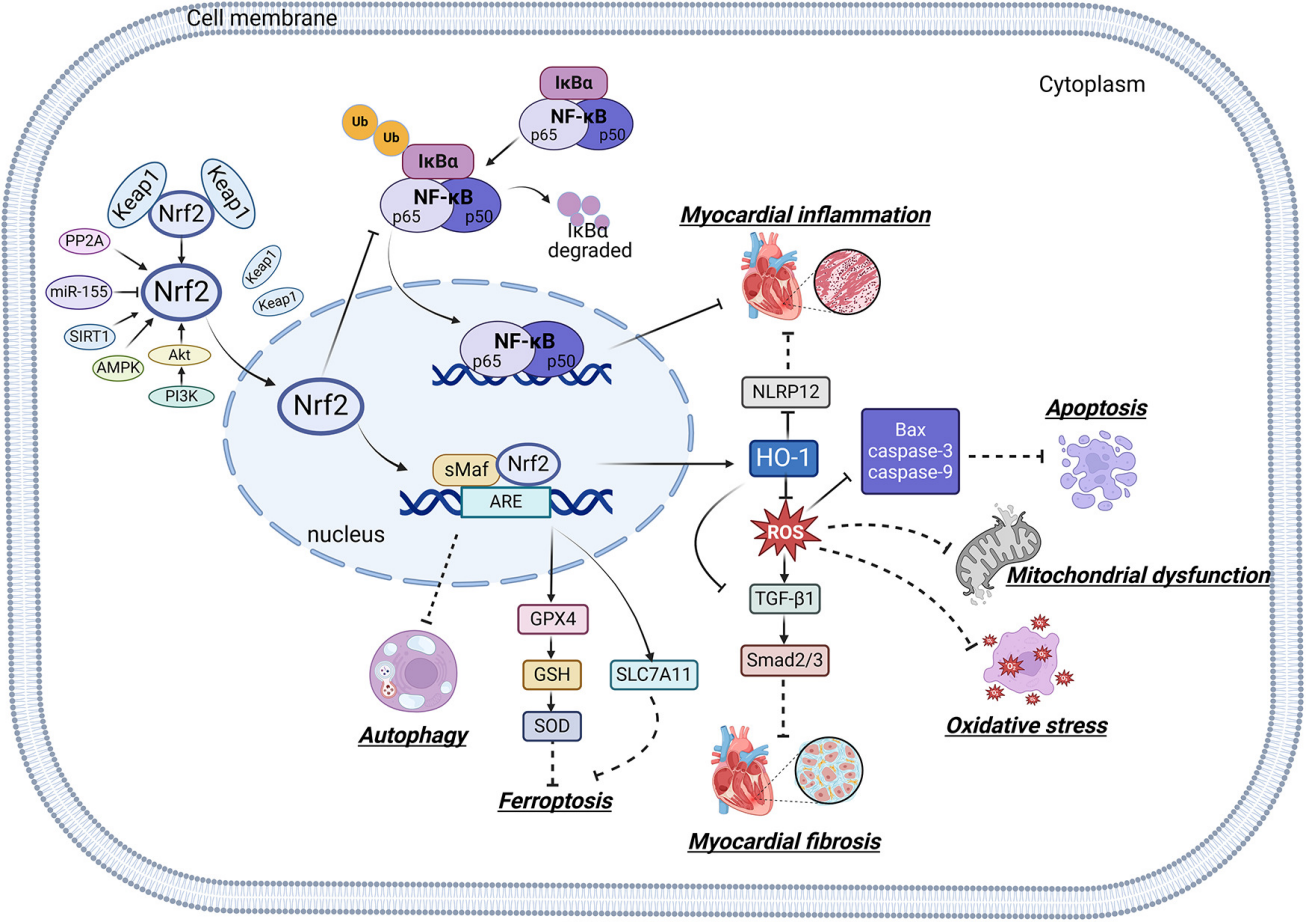
Relationship between Nrf2 signaling pathway and oxidative stress, inflammation, myocardial fibrosis, apoptosis, ferroptosis, autophagy and mitochondrial dysfunction.

Mitochondria are crucial organelles in cardiomyocytes responsible for energy supply. Studies have demonstrated that the production of adenosine 5′-triphosphate (ATP) by mitochondria is accompanied by the generation of ROS. Under normal physiological conditions, cardiomyocytes activate antioxidant defense mechanisms to counteract ROS damage. However, mitochondrial dysfunction in metabolic disorders can disrupt the respiratory chain, leading to excessive ROS production in the heart. The accumulation of ROS not only impairs mitochondrial structure and function but also promotes lipid buildup. These effects may contribute to myocardial fibrosis and cardiac diastolic dysfunction in the progression of DCM (70–72). Therefore, mitochondrial dysfunction is considered a key factor of oxidative stress. Sustained hyperglycemia acts as the primary driver of mitochondrial impairment in cardiomyocytes (73, 74). According to research findings, impairment of mitochondrial respiratory function, mitochondrial membrane potential and mitochondrial biogenesis in the hearts of diabetic rats contributes to atrial structure remodeling and alterations in electrical activity, thereby facilitating the onset of atrial fibrillation (75). Knockout of Nrf2 gene significantly impairs mitochondrial respiratory function and reduces mitochondrial membrane potential and decreases ATP production in cardiomyocytes of db/db mice (76). Conversely, activation of the Nrf2/HO-1 signaling pathway improves mitochondrial dysfunction in cardiomyocytes (77). In diabetic rats, activation of the SIRT1/Nrf2/HO-1/Nox-2 pathway effectively alleviates mitochondrial dysfunction and oxidative stress in cardiomyocytes. This is achieved by reducing ROS production, increasing ATP levels and enhancing the activity of mitochondrial enzymes in myocardial tissues. Ultimately, these improvements contribute to the restoration of cardiac function in diabetic rats (78). Therefore, the activation of Nrf2 may represent a promising therapeutic target for the treatment of diabetic cardiomyopathy by restoring mitochondrial function (Figure 2).

## 3 Traditional Chinese medicine for the treatment of DCM through the activation of Nrf2 signaling pathway

Pharmacological NRF2 activators have shown significant protective effects across a range of disease models and have yielded promising results in human intervention trials, thereby solidifying NRF2 as a highly promising drug target (79). Existing evidences have confirmed that some specific TCMs and their bioactive components which have the potential efficacy of DCM are associated with the activation of Nrf2 signaling pathway. In this section, we presented a comprehensive overview of the effects and mechanisms of these TCMs and their bioactive ingredients in the context of DCM.

### 3.1 Bioactive compounds of Nrf2 agonists from TCM

#### 3.1.1 Resveratrol

The natural polyphenol compound of resveratrol (RES) was originally derived from *Veratrum grandiflorum* (Maxim. ex Baker) Loes., *Polygonum cuspidatum* Sieb. et Zucc., *Cassia obtusifolia* L. and mulberry. In the past 20 years, nearly 200 clinical studies have evaluated RES for 24 indications, including cancer, menopause symptoms, diabetes, metabolic syndrome, and cardiovascular disease (80). Clinical trials confirm that RSV significantly improves left ventricular function and reduces premature atrial and ventricular contractions with decreasing the serum of aspartate aminotransferase, glucose, LDL cholesterol, alanine aminotransferase, total cholesterol and insulin resistance index (81, 82). It is suggested that RES, as a form of Nrf2 agonists has been shown the promising evidence of efficacy on DCM therapy both in clinical trials and lab experiments. Furthermore, numerous studies have demonstrated that RES exerts its antioxidant effects and anti-inflammatory and cardiovascular protective effects through the Nrf2 signaling pathway. It has been shown that RES protects the hyperglycemia-induced cardiomyocytes by promoting the Nrf2 protein expression and its downstream of antioxidant genes (83). Besides, intraperitoneal administration of RES for type 2 diabetic rats reduces myocardial ischemia/reperfusion injury (MIRI) by activating the AMPK/p38/Nrf2 signaling pathway (84, 85).

#### 3.1.2 Quercetin

QUR, a natural flavonoid possessing antioxidant, anti-inflammatory, anti-atherosclerotic, anti-thrombotic and cardioprotective activities is widely distributed in Chinese herbal medicines, fruits, leaves, vegetables, seeds and plant roots (86, 87). Quercetin supplementation demonstrates moderate-to high-quality evidence for reducing cardiovascular disease (CVD) risk factors. Multiple randomized controlled trials examine the effects of quercetin on patients with coronary artery disease and demonstrate a significant reduction in chronic systemic inflammation (88). Priclincal studies have shown that QUR protects the myocardium from MIRI by inhibiting the inflammatory cascade and apoptosis via the PI3K/Akt signaling pathway (88). QUR also alleviates oxidative stress by enhancing the level of SOD, CAT and GPx, while mitigating inflammation and apoptosis through down-regulating the expression levels of IL-6 and Bax in cardiomyocytes of STZ-nicotinamide-induced diabetic rats. The Nrf2 signaling pathway may be a target for DCM therapy (89). In diabetic rats model, QUR reduces the accumulation of ROS in cardiomyocytes and delays the development of myocardial fibrosis by facilitating the nuclear translocation of Nrf2, which subsequently increases the expression levels of its downstream target genes including HO-1, SOD and glutamate-cysteine ligase catalytic (GCLC) (90). QUR has also been found to ameliorate hyperglycemia-induced myocardial bioenergetic damage and maintain intracellular energy homeostasis in diabetic rats by upregulating the expression levels of Nrf2, HO-1, SOD and proliferator-activated receptor gamma coactivator-1α (PGC-1α) (91, 92).

#### 3.1.3 Curcumin

Curcumin (CUR), a naturally occurring polyphenolic compound derived from medicinal plants such as *Curcuma wenyujin* Y.H., Chen et C. Ling, *Curcuma longa* L., and *Curcuma phaeocaulis* Vai. (family Zingiberaceae), exhibits the antioxidant, anti-inflammatory, anticancer and antiapoptotic activities (93, 94). A randomized clinical trial suggests that curcumin significantly enhances both insulin sensitivity and the homeostatic model assessment of insulin resistance (HOMA-IR) (95). Preclincal study has confirmed that CUR has protective effects on DCM via the Nrf2-related signaling pathway (96). In high glucose-induced H9c2 cells and cardiomyocytes from type 2 diabetic rats, CUR increases cardiomyocyte viability and antioxidant enzyme activity, reduces ROS formation and cardiomyocyte apoptosis through activation of the Nrf2/HO-1 signaling pathway (97). Additionally, researchers have shown that CUR exerts protective effects against oxidative stress and ferroptosis-induced injury in the cardiomyocytes of diabetic rats by promoting Nrf2 nuclear translocation and upregulating the expression of its downstream genes (59, 98). CUR also can reduce the accumulation of superoxide and inhibit pyroptosis in the cardiomyocytes of diabetic rats through the activation of the AKT/Nrf2/ARE pathway (99).

#### 3.1.4 Sulforaphane

Sulforaphane (SFN), a natural compound from the herb extracts belonging to the *Cruciferae* family (100) is one of the first identified and most potent naturally occurring Nrf2 activators (101). The overall outcomes of the clinical trials with sulforaphane-rich preparations have reinforced the preclinical evidence that sulforaphane has the potential to ameliorate a variety of diseases related to chronic metabolic and inflammatory stress (102, 103). A diversity preclinical experiments also shown improvement effect of sulforaphane on DCM. Studies have confirmed that SFN prevents cardiomyocyte oxidative damage, inflammation, and fibrosis in diabetic mouse models through the activation of the AMPK/AKT/GSK3β/NRF2 signaling pathway (104, 105). Furthermore, SFN alleviates cardiomyocyte hypertrophy and fibrosis in DCM mice through upregulating the expression and transcriptional activity of Nrf2. Silencing the Nrf2 gene in high glucose-induced H9c2 cells abolishes the protective effect of SFN on cardiomyocyte fibrosis (106). Moreover, SFN can prevent the cardiomyocyte ferroptosis in DCM mice model through activation of the AMPK/Nrf2 signaling pathway (63). These results suggest that Nrf2 and its related signaling pathway may be the key targets of SFN for treating DCM.

#### 3.1.5 Luteolin

Luteolin (LUT) as a natural antioxidant is widely found in fruits, vegetables, flowers and herbs with excellent radical scavenging and cytoprotective properties. LUT is emerging as one of the most promising candidates in the biomedical and pharmaceutical fields (107). Recent studies have reported that LUT exhibits cardioprotective effects both *in vitro* and *vivo* (108). Researchers have confirmed that LUT has a protective effect on DCM via the Nrf2 signaling pathway. It has been proven that LUT ameliorates cardiac function and myocardial viability in diabetic rats with ischemia/reperfusion injury through the Nrf2-regulated antioxidative signaling pathway (109, 110). Evidence has demonstrated that LUT suppresses cardiomyocyte inflammation and oxidative stress, thereby preventing myocardial fibrosis and hypertrophy in DCM mice model via the upregulation of Nrf2, HO-1, and NQO1 (111). Although there were limited clinical trials correlated with the therapeutic effect of LUT on DCM, the exploration of its therapeutic effects on human DCM and related mechanisms targeting Nrf2 will become a hot topic among researchers.

#### 3.1.6 Kaempferol

Kaempferol (KMP) as a plant-derived flavonoid has various pharmacological activities such as antioxidant, anti-inflammatory, anticancer and cardioprotective effects (112). KMP-containing plants are used worldwide in traditional systems to treat various conditions for centuries (113). Research has revealed that KMP as one of the main compounds of *Eucomm*iae Folium exerted the cardioprotective effect on DCM mice (114). What's worth noting that KMP attenuates oxidative, inflammatory and fibrotic damages of the left ventricle (LV) in STZ-induced diabetic rats by upregulating the SIRT1/Nrf2 signaling pathway (115). KMP also protects against isoproterenol (ISO) -induced heart failure in diabetic rats by inhibiting cardiomyocyte apoptosis and activating the PI3K/Akt/GSK-3β/Nrf2 signaling pathway (116). Additionally, KMP as the Nrf2 activator demonstrates the beneficial effects on cardiac structure and function and its prominent anti-cardiac remodeling properties by inhibiting inflammatory responses and oxidative stress expression both *in vitro* and vivo DCM model (117). However, clinical studies are needed to confirm the protective effects of kaempferol observed in laboratory settings.

#### 3.1.7 Notoginsenoside R1

*Panax notoginseng* (PN) root serves as a widely recognized nutritional supplement, health food ingredient, and traditional medicine. It plays a crucial role in maintaining homeostasis within the human microcirculatory system. Notoginsenoside R1 (NGR1), an active compound derived from *Panax notoginseng* (PN) root, has been reported to exhibit a range of pharmacological activities, including anti-inflammatory, antioxidant, anticancer, antimicrobial and angiogenic effects (118, 119). Clinical studies have demonstrated the efficacy of incorporating NGR1, the primary bioactive component of the XueShuanTong formula, into conventional treatments for ischemic diseases (120). Preclincal study has indicated that NGR1 prevents vascular smooth muscle cell (VSMC) proliferation, migration and neointimal proliferation by inhibiting activation of the PI3K/Akt signaling pathway (121). Recent experiment has confirmed that NGR1 inhibits cell apoptosis and hypertrophy by upregulating the AMPK/Nrf2 signaling pathway and HO-1 expression. The levels of cardiac hypertrophy markers, including auricular natriuretic peptide (ANP) and brain natriuretic peptide (BNP) are significantly decreased (122).

#### 3.1.8 Rg1 ginsenoside

Ginsenoside Rg1 (GRg1) is a primary active component of *Panax ginseng* C.A. Mey. It has been shown to have a protective effect on various cardiovascular diseases by regulating multiple cellular signaling pathways (123, 124). GRg1 has been proven to protect against DCM. Studies have demonstrated that GRg1 ameliorates cardiomyocyte oxidative stress and inflammation in DCM rats through activation of the AMPK/Nrf2/HO-1 signaling pathway (125). However, the precise role of GRg1 in regulating the Nrf2 signaling pathway within the treatment of DCM deserves further investigation.

#### 3.1.9 Myricetin (杨梅素) and myricitrin (杨梅苷)

Myricetin, a flavonoid compound derived from various fruit, vegetables, tea, berries and red wine (126). Myricetin displays multiple preclinical biological effects including antioxidant, anti-inflammatory, anticancer, antidiabetic, antiviral, antibacterial and cardiovascular protective effects (127). Besides, clinical trials from various studies highlight the importance of myricetin as a chemo preventive reagent and its significant positive impact on key risk factors for coronary heart disease (128). In both lipopolysaccharide (LPS) -induced H9c2 cells and a C57BL/6J diabetic mouse model, myricetin mitigates cardiomyocyte oxidative stress and inflammation injuries (129). Studies have shown that myricetin alleviates pressure overload-induced cardiac hypertrophy in Nrf2 knockdown (Nrf2-KD) mice and phenylephrine (PE)-induced neonatal rat cardiomyocytes (NRCMs) via activation of the Nrf2 signaling pathway (130). Additionally, myricetin attenuates oxidative stress, inflammation and apoptosis in cardiomyocytes and improves myocardial diastolic dysfunction in STZ-induced diabetic mice through upregulation of the Nrf2/HO-1 signaling pathway (131, 132).

#### 3.1.10 Naringenin (柚皮素) and naringin (柚皮苷)

**Figure 3:**
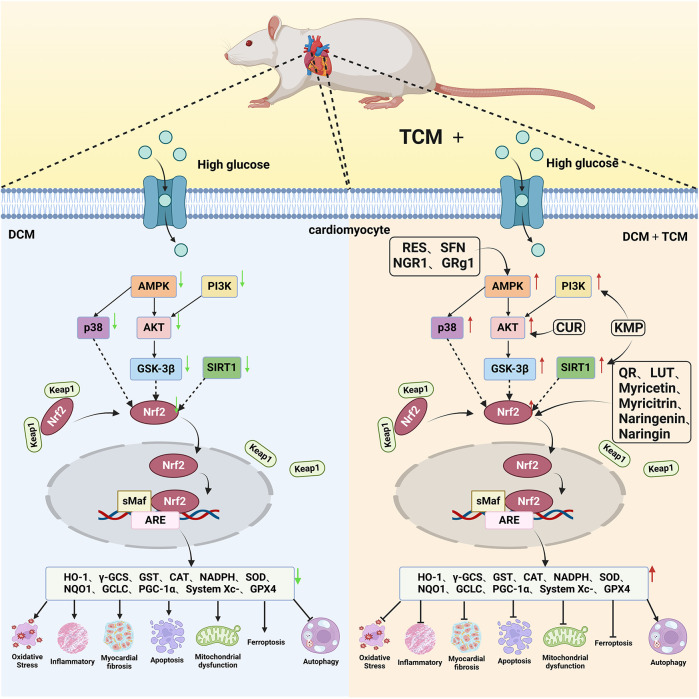
The cardioprotective effect of TCM on DCM rats via Nrf2 signaling pathway. RES, resveratrol SFN, sulforaphane; NGR1, Notoginsenoside R1; GRg1, Ginsenoside Rg1; CUR, curcumin; KMP, kaempferol; QR, quercetin; LUT, luteolin.

Naringin, a flavanone glycoside exists in two forms: the glycosidic form of naringin and the aglycone form of naringenin (133). As flavonoids, naringenin and naringin possess a diverse range of pharmacological activities including antioxidant, anti-inflammatory, antidiabetic, anticancer and cardiovascular disease prevention effects (134). Naringenin exhibites beneficial effects on the lipid profile and reduces the percentages of non-alcoholic fatty liver disease (NAFLD) grades, serving as an indicator of the severity of hepatic steatosis for NAFLD patients. Clinical trials demonstrate that naringenin has a beneficial effect particularly related to cardiovascular diseases and diabetes (135). Preclinical study indicates that naringenin alleviates pathological damage, inflammation, lipid peroxidation and cellular ferroptosis by modulating the Nrf2/System Xc-/GPX4 axis in the myocardial tissue of MIRI-induced rats (136). Additionally, research has revealed that the protective effect of naringin on cardiomyocytes in diabetic mice which may be associated with reducing intracellular Ca^2+^ overload, limiting the increase in ROS levels and suppressing the expression level of TNF-α, IL-6, and NF-κB (137). Studies have also demonstrated that both naringenin and naringin ameliorate cardiomyocyte oxidative stress, inflammation and apoptosis in STZ-induced diabetic mice through activation of the Nrf2 signaling pathway (138, 139). Consequently, the preclinical studies indicate that Nrf2 may serve as a potential target for naringenin and naringin in the prevention of DCM (Figure 3).

The next section provided a review of the protective effects of representative natural compounds on DCM and their potential mechanisms related to the Nrf2 signaling pathway, as detailed in Table 1.

**Table 1:** Effects of Chinese medicine compounds on Nrf2 signaling pathway and their roles in DCM.

NO.	Chinese Medicine Monomer	Chemical Structural Formula	Model	Regulation of Nrf2 Signaling Pathway	Main Purposed Effects	Ref(s)
1	Resveratrol	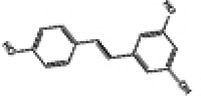	FVB mice	↑Nrf2, HO-1, SOD, NADPH	Anti-oxidative stress	(83–85)
					Anti-myocardial fibrosis	
			SD rats		Anti-myocardial hypertrophy	
2	Quercetin	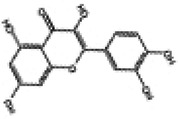	SD rats	↑Nrf2, HO-1, SOD, GSH	Anti-oxidative stress	(90, 91)
			H9c2 cells			
					Anti-myocardial fibrosis	
			Wistar rats			
3	Curcumin	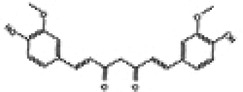	SD rats	↑Nrf2, HO-1, GSH	Anti-oxidative stress	(59, 97–99)
			H9c2 cells		Anti-cardiomyocyte apoptosis	
			New Zealand rabbits		Anti-cardiomyocyte ferroptosis	
			Wistar rats		Anti-inflammation	
4	Sulforaphane	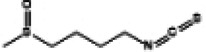	FVB mice	↑Nrf2, HO-1, SOD, MT, CAT	Anti-oxidative stress	(63, 104–106)
					Anti-myocardial fibrosis	
					Anti-inflammation	
			C57BL/6J mice		Anti-myocardial fibrosis	
					Anti-myocardial hypertrophy	
5	Luteolin	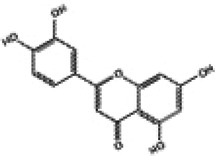	SD rats	↑Nrf2, HO-1, SOD, GPx	Anti-oxidative stress	(109–111)
			H9c2 cells		Anti-inflammation	
			C57BL/6J mice		Anti-myocardial fibrosis	
					Anti-myocardial hypertrophy	
					Anti-mitochondrial damage	
6	Kaempferol	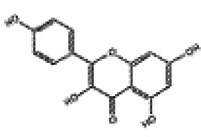	Wistar rats	↑Nrf2, HO-1, *γ*-GCS	Anti-oxidative stress	(115–117)
			C57BL/6J mice		Anti-inflammation	
					Anti-myocardial fibrosis	
			H9c2 cells			
					Anti-cardiomyocyte apoptosis	
7	Notoginsenoside R1	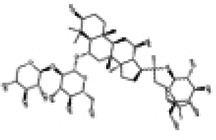	H9c2 cells	↑Nrf2, HO-1	Anti-oxidative stress	(122)
					Anti-cardiomyocyte apoptosis	
8	Ginsenoside Rg1	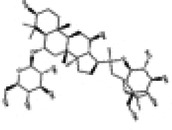	Wistar rats	↑Nrf2, HO-1, SOD, CAT	Anti-oxidative stress	(125)
					Anti-inflammation	
9	Myricetin	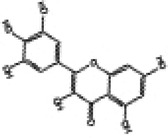	C57BL/6J mice	↑Nrf2, HO-1, SOD, NQO1	Anti-oxidative stress	(131)
					Anti-inflammation	
					Anti-cardiomyocyte apoptosis	
10	Myricitrin	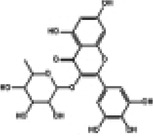	BALB/c mice	↑Nrf2, HO-1, NQO-1	Anti-oxidative stress	(132)
					Anti-inflammation	
			H9c2 cells		Anti-cardiomyocyte apoptosis	
					Anti-myocardial fibrosis	
11	Naringenin	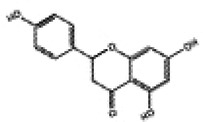	C57BL/6J mice	↑Nrf2, HO-1, SOD, NQO-1	Anti-oxidative stress	(138)
					Anti-inflammation	
			H9c2 cells		Anti-cardiomyocyte apoptosis	
12	Naringin	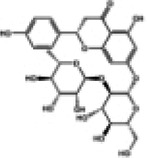	SD rats	↑Nrf2	Anti-oxidative stress	(139)
13	Thymoquinone	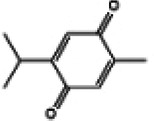	Wistar rats	↑Nrf2, HO-1, SOD	Anti-oxidative stress	(140)
					Anti-inflammation	
14	Bakuchiol	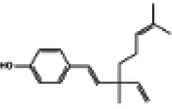	C57BL/6J mice	↑Nrf2, SOD, GPx	Anti-oxidative stress	(141)
					Anti-myocardial fibrosis	
			H9c2 cells		Anti-myocardial hypertrophy	
					Anti-cardiomyocyte apoptosis	
15	Andrographolide	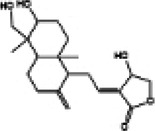	C57BL/6J mice	↑Nrf2, HO-1, SOD	Anti-oxidative stress	(142)
			H9c2 cells		Anti-inflammation	
					Anti-cardiomyocyte apoptosis	
16	Dimethyl fumarate	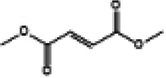	C57BL/6J mice	↑Nrf2, HO-1, SOD, CAT	Anti-oxidative stress	(143)
					Anti-inflammation	
					Anti-myocardial fibrosis	
17	Glycyrrhizin	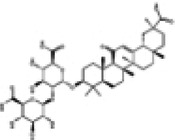	Zucker Diabetic Fatty rats	↑Nrf2	Anti-oxidative stress	(144)
					Anti-inflammation	
					Anti-myocardial fibrosis	
			AC16 human cardiomyocyte			
18	Scutellarin	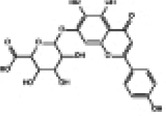	C57BL/6J mice	↑Nrf2, HO-1, SOD, CAT	Anti-oxidative stress	(145)
					Anti-inflammation	
					Anti-myocardial fibrosis	
19	Bail calin	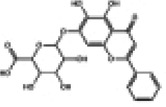	C57BL/6J mice	↑Nrf2, HO-1, NQO1	Anti-inflammation	(146)
					Anti-myocardial fibrosis	
					Anti-myocardial hypertrophy	
					Anti-cardiomyocyte apoptosis	
20	Honokiol	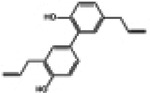	SD rats	↑Nrf2, HO-1, NQO1	Anti-oxidative stress	(147)
			H9c2 cells		Anti-cardiomyocyte apoptosis	
21	Cyclovirobuxine D	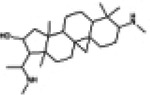	SD rats	↑Nrf2, NQO1	Anti-oxidative stress	(148)
			The primary neonatalrat cardiomyocyte			
22	Sinapic acid	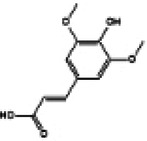	Wistar rats	↑Nrf2, HO-1	Anti-oxidative stress	(149)
					Anti-inflammation	
					Anti-cardiomyocyte apoptosis	
23	Oleanolic acid	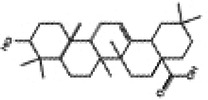	SD rats	↑Nrf2, HO-1	Anti-oxidative stress	(150)
					Anti-cardiomyocyte apoptosis	
24	Fucoxanthin	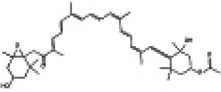	SD rats	↑Nrf2, HO-1, SOD	Anti-oxidative stress	(151)
					Anti-myocardial fibrosis	
					Anti-myocardial hypertrophy	
25	6-Gingerol	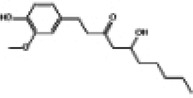	C57BL/6J mice	↑Nrf2, HO-1	Anti-oxidative stress	(152)
					Anti-inflammation	
			H9c2 cells		Anti-cardiomyocyte ferroptosis	
					Anti-cardiomyocyte apoptosis	
26	Piceatannol	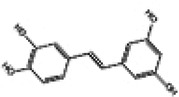	SD rats	↑Nrf2, HO-1	Anti-oxidative stress	(153)
					Anti-inflammation	
			H9c2 cells		Anti-cardiomyocyte ferroptosis	
					Anti-cardiomyocyte apoptosis	
27	Fortunellin	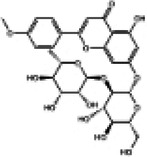	C57BL/6J mice	↑Nrf2, HO-1, SOD, CAT	Anti-oxidative stress	(154)
			H9c2 cells		Anti-inflammation	
28	Costunolide	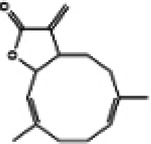	C57BL/6J mice	↑Nrf2, HO-1	Anti-oxidative stress	(155)
					Anti-inflammation	
			H9c2 cells		Anti-cardiomyocyte ferroptosis	
					Anti-myocardial hypertrophy	
29	Geniposide	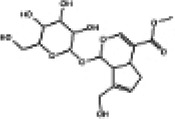	SD rats	↑Nrf2, HO-1	Anti-oxidative stress	(156)
30	Butin	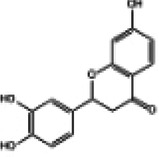	C57BL/6J mice	↑Nrf2, HO-1	Anti-oxidative stress	(157)
			H9c2 cells			
31	Kolaviron	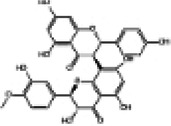	SD rats	↑Nrf2, SOD	Anti-oxidative stress	(158)
					Anti-inflammation	
32	Diallyl trisulfide	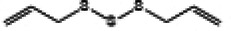	SD rats	↑Nrf2, HO-1	Anti-oxidative stress	(159)
33	Phloretin	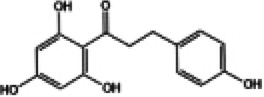	C57BL/6J mice	↑Nrf2, HO-1, SOD, NQO1	Anti-oxidative stress	(160)
					Anti-myocardial fibrosis	
					Anti-myocardial hypertrophy	
34	Gastrodin	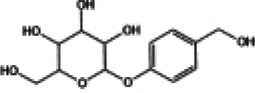	H9c2 cells	↑Nrf2, GSH, SOD, CAT	Anti-oxidative stress	(161)
35	Esculeoside A	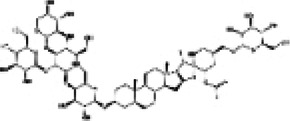	H9c2 cells	↑Nrf2, OH-1, GSH, SOD	Anti-oxidative stress	(162)
					Anti-inflammation	
					Anti-cardiomyocyte apoptosis	
36	Ginsenoside Rb1	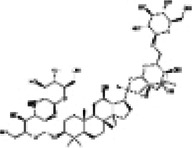	Wistar rats	↑Nrf2, HO-1, SOD, CAT	Anti-oxidative stress	(163)
					Anti-myocardial fibrosis	
37	Asiaticoside	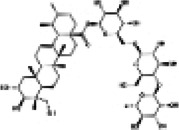	db/db mice	↑Nrf2, HO-1	Anti-oxidative stress	(164)
38	Pterostilbene	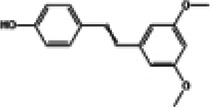	SD rats	↑Nrf2, HO-1	Anti-oxidative stress	(165)
					Anti-inflammation	

↑ signifies increase/activation.

### 3.2 Nrf2 agonists from Chinese herbal prescriptions

In China, various medicinal plants are combined as TCM prescriptions for the treatment of diseases. TCM prescriptions known for their multi-target, multi-component and multi-pathway characteristics, exerts synergistic effects on DCM therapy. In this section, we reviewed representative TCM prescriptions for DCM treatment that were associated with the Nrf2 signaling pathway.

#### 3.2.1 Guan Xin Dan Shen formulation

The Guan Xin Dan Shen Formulation (GXDSF) consists of three herbs: *Dalbergia odoriferae* Lignum, *Salviae miltiorrhizae* Radix et Rhizoma, and *Panax notoginseng* Radix et Rhizoma. It has been widely used for the management of coronary heart disease in China by activating blood circulation, resolving blood stasis and relieving pain (166). GXDSF has been used clinically for the treatment of cardiovascular diseases (23). The main active ingredients of GXDSF are ginsenoside Rg1 (9.51%), ginsenoside Rb1 (8.63%), notoginsenoside R1 (2.34%), tanshinone IIA (1.71%), cryptotanshinone (0.84%), tanshinone I (0.55%) and salvianolic acid B (0.50%) (167). Salvianolic acid B and notoginsenoside R1 in GXDSF have been shown to significantly protect H9c2 cardiomyocytes from hypoxia and reoxygenation injuries by reducing the levels of the inflammatory factors of TNF-α and IL-1β (168). Preclinical studies indicate that treatment with GXDSF improves cardiac hypertrophy and dysfunction and significantly increases the left ventricular ejection fraction (LVEF) in diabetic mice model. Additionally, GXDSF attenuates cardiac dysfunction and inhibits cardiomyocyte apoptosis by activating the Akt/Nrf2 signaling pathway (169).

#### 3.2.2 Mulberry granules

Mulberry granules a traditional Chinese medicine prescription is derived from the fruit of *Morus alba* L (170). It contains various beneficial constituents especially flavonoids and alkaloids (171, 172). In China, mulberry is commonly used to treat diabetes palpitations, insomnia and hyperglycemia for many years (173). Clinical studies have confirmed that mulberry twig alkaloids are effective and safe for the treatment of type 2 diabetes (174). Besides, including mulberry in the diet could positively influence various cardiometabolic risk factors (175). Preclinical experiment indicates that mulberry has been found to improve insulin sensitivity by activating the AMPK signaling pathway in diabetic db/db mice (176). Research has shown that mulberry ethanol extracts ameliorate abnormal lipid metabolism and enhance antioxidant activity in atherosclerosis (AS) rats (177). It can be speculated that mulberry could be benefit for DCM. Furthermore, mulberry attenuates oxidative stress induced by myocardial ischemia-reperfusion injury through upregulating the expression of GSH, SOD, CAT and glutathione reductase (GR) in myocardial tissue via the AMPK/Nrf2 signaling pathway (178). These preclinical studies suggest that mulberry granules may exert protective effects on DCM through the AMPK/Nrf2 signaling pathway. However, further clinical and animal studies are required to investigate the mechanism by which mulberry granules influences the progression of DCM.

#### 3.2.3 Polyherbal formulation

The polyherbal formulation (PHF) comprises *Piper nigrum* (fruit), *Terminalia paniculata* (bark) and *Bauhinia purpurea* (bark), three of which are ayurvedic medicines and is used India's conventional medicinal system. Based on animal experiments, researchers shown that PHF reduces oxidative stress and inflammation in cardiac tissues of diabetic rats. This mechanism may be associated with the upregulation of the Nrf2/HO-1 signaling pathway. Furthermore, PHF increases the serum levels of SOD, CAT and GSH and decreases the serum levels IL-1β, IL-6 and TNF-α, which is closely associated with the activation of the NF-κB/Nrf2/HO-1 signaling pathway (179). These findings demonstrate that Nrf2 may be considered as the key targets of PHF for preclinical treatment of DCM.

#### 3.2.4 Benefits and challenges of TCMs as Nrf2 activators for DCM therapy

Due to the oxidative stress as an essential pathogenic factor for DCM pathogenesis, the TCMs as Nrf2 activators exert protective effects for cardiovascular tissues in diabetes. Aforementioned evidences reveal that mechanism and potential of the TCMs against DCM are prominent in the PI3K/Akt/GSK-3β, NF-κB, AMPK/p38, SIRT1 and TGF-β/Smads signaling pathway which are crosstalk with the activation of Nrf2. These evidences also support the conclusion that the TCM alleviates DCM by modulating pathological processes, including myocardial fibrosis, inflammation, oxidative stress, metabolism disorder, cardiac hypertrophy, apoptosis and etc. Considering the damage to multiple organs caused by hyperglycemia as well as the intricate and prolonged development of DCM, TCMs as the Nrf2 activators demonstrate significant promise as a potential option for DCM due to its advantages of multi-target and multi-pathway effects with limited adverse reactions. However, numerous challenges remain in elucidating both the biological activity and potential toxicity of TCMs for their future clinical application in DCM. Additionally, the larger clinical trials of the Nrf2 activators from TCMs are urgently needed to further evaluation the reliability of their therapeutic effects.

## 4 Future prospects

The pathological mechanism of DCM involves in the interplay of various molecular signal transduction pathways. Nrf2 as a key transcription factor in the pathogenesis of DCM provides protection effects by reducing oxidative stress, inflammation, myocardial fibrosis, apoptosis, ferroptosis, autophagy and mitochondrial dysfunction. However, there are still unexplored pathways or processes linked to Nrf2 that need further explained its role in DCM. Copper (Cu) regulation in the pathogenesis of DCM has been drawn attention as a new research hotspot. Researcher speculates that in the progression of DCM, the high levels of Cu in plasma may damage mitochondria of vascular endothelial cells through cuproptosis or oxidative stress pathway and then affects the diastolic and contractile function of cardiomyocytes (180). While Cu deficiency in myocardial cells may result in impairment of energy metabolism (181). Animal study confirms that Cu deficiency can inhibit the Nrf2 pathway, consequently induces oxidative damage in the liver (182). However, the exact mechanism by which Cu affects antioxidant related to regulation of Nrf2 deserves as another interesting research.

This review systematically analyses the role of the Nrf2 signaling pathway in the pathogenesis and treatment of DCM using TCM. Based on the literature, it can be concluded that Nrf2 as a classical transcription factor associated with anti-oxidative stress is a promising target for DCM treatment. However, the role of Nrf2 in DCM also represents complex and controversial aspect. One recent study has found that chronic Nrf2 activation in the context of autophagy deficiency can exacerbate DCM, suggesting that Nrf2 is not universally beneficial (183). Additionally, although TCM formulations have shown efficacy in ameliorating and treating DCM potentially by targeting the Nrf2 signaling pathway in preclinical experiments, further exploration of clinical researches is still required to validate the safety and efficacy of TCM formulations for DCM treatment. Furthermore, the majority studies on compounds from TCM have primarily focused on animal and cell experiments. How to translate these findings into clinical practice for DCM faces several potential limitations and challenges. Firstly, limitations of study methodology. Many studies focus on *in vitro* experiments, which may differ from the *in vivo* conditions. Although animal experiments are crucial for understanding TCM's mechanisms, it is different from human beings in terms of anatomy, physiology and metabolism. Secondly, the complexity of TCM components propose the challenges for revealing the precise molecular mechanism for treatment of DCM, which hinders their global recognition. TCM's complex composition makes it difficult to identify the major active ingredients. Thirdly, the clinical detection of Nrf2 presents another significant challenge. Although Nrf2 has been generally accepted as one of key anti-oxidative transcription factor in the progression of DCM, it has yet to be established as a reliable biomarker for both the diagnosis and treatment of DCM in clinical practice. Other possible diagnostic markers that indirectly reflect the Nrf2 activity needs to be explored. Nrf2 signaling, a key driver of antioxidation, is commonly down-regulated in DCM. Current TCM targeting the Nrf2 signaling pathway has been shown promise in preclinical trials. Further improvement understanding of the regulatory mechanisms of Nrf2 signaling and extensive clinical trial studies of natural Nrf2 activators will provide new approaches for the treatment of DCM patients in the near future.
